# A Novel Method to Generate and Expand Clinical-Grade, Genetically Modified, Tumor-Infiltrating Lymphocytes

**DOI:** 10.3389/fimmu.2017.00908

**Published:** 2017-08-02

**Authors:** Marie-Andrée Forget, René J. Tavera, Cara Haymaker, Renjith Ramachandran, Shuti Malu, Minying Zhang, Seth Wardell, Orenthial J. Fulbright, Chistopher Leroy Toth, Audrey M. Gonzalez, Shawne T. Thorsen, Esteban Flores, Arely Wahl, Weiyi Peng, Rodabe N. Amaria, Patrick Hwu, Chantale Bernatchez

**Affiliations:** ^1^Department of Melanoma Medical Oncology, The University of Texas MD Anderson Cancer Center (MDACC), Houston, TX, United States

**Keywords:** tumor-infiltrating lymphocytes, retroviral-transduction, adoptive cell therapy, genetic modification, Good Manufacturing Practice, clinical-grade

## Abstract

Following the clinical success achieved with the first generation of adoptive cell therapy (ACT) utilizing *in vitro* expanded tumor-infiltrating lymphocytes (TILs), the second and third generations of TIL ACT are evolving toward the use of genetically modified TIL. TIL therapy generally involves the transfer of a high number of TIL, ranging from 10^9^ to 10^11^ cells. One of the technical difficulties in genetically modifying TIL, using a retroviral vector, is the ability to achieve large expansion of transduced TIL, while keeping the technique suitable to a Good Manufacturing Practices (GMP) environment. Consequently, we developed and optimized a novel method for the efficient production of large numbers of GMP-grade, gene-modified TIL for the treatment of patients with ACT. The chemokine receptor CXCR2 was used as the gene of interest for methodology development. The optimized procedure is currently used in the production of gene-modified TIL for two clinical trials for the treatment of metastatic melanoma at MD Anderson Cancer Center.

## Introduction

Adoptive cell therapy (ACT) utilizing tumor-infiltrating lymphocytes (TIL) has demonstrated clinical success in the treatment of metastatic melanoma in studies at MD Anderson Cancer Center (MDACC) as well as in multiple studies performed worldwide (1–4). The success of TIL ACT encompasses two factors: the diverse antigenic specificity toward the autologous tumor displayed by TIL and their lytic capability to eradicate the tumor. We have reported that response to therapy positively correlates with the total number of TIL infused as well as the number of CD8^+^ TIL infused (2). However, within the success of TIL ACT, there still lies different challenges to overcome in order to improve this ultimate personalized therapy. Such challenges are comprised of the tumor microenvironment itself which is difficult to infiltrate and overwhelmed with immune-suppressive mechanisms. Preclinical work by our group and others has shown that TIL could be genetically modified to either facilitate their entry into the tumor using the chemokine receptor CXCR2 or protect them against immune-suppression by introducing a dominant negative form of the TGF-βRII with both modifications displaying an improved *in vivo* antitumor activity (5–7). Thus, it became evident that TIL, a T-cell product enriched for antitumor reactivity, represented the best platform to test functional enhancements derived from gene-modification in the clinic. We committed to develop a TIL transduction protocol that would not compromise the yield of cells generated and that is suitable for use in a Good Manufacturing Practices (GMP) suite.

The feasibility of genetically modifying human TIL using a retrovirus platform, and its safety in the treatment of metastatic melanoma was demonstrated over two decades ago (8). Other clinical trials later followed, all seeking to implement improvements to TIL ACT (9, 10). The total number of TIL infused to patients on these gene-modified TIL studies was low (1–5 billion) of which only a portion expressed the transduced gene.

One of the technical difficulties in genetically modifying TIL for a clinical large-scale expansion is the ability to achieve the target expansion, while keeping the technique suitable to a GMP environment. Consequently, we developed a novel method in which we have addressed the formerly described challenges, and optimized the conditions for the efficient gene-modification and production of GMP-grade TIL for the treatment of patients with ACT. In this method, the chemokine receptor CXCR2 was used as the gene of interest to be optimized for transduction. CXCR2 is one of the two candidate genes for which a clinical trial of gene-modified TIL for patients with metastatic melanoma was designed at MDACC. This study describes the process used to develop this novel method to genetically modify and expand GMP clinical-grade TIL.

## Materials and Methods

### Normal Donor and Patient Population

Peripheral blood mononuclear cells (PBMCs) from normal donors were obtained from buffy coats purchased from the Gulf Coast Regional Blood Center (Houston, TX, USA). TIL lines used for experimental validation were derived from tumor tissue obtained from patients with metastatic melanoma enrolled on a TIL ACT clinical trial [Institutional review board (IRB)-approved protocol# 2004-0069, NCT00338377] at the University of Texas MDACC. Male and female patients over the age of 12 with stage IV melanoma, stage III in-transit disease, recurrent regional nodal disease or uveal melanoma were eligible for enrollment. Refer to clinical trial NCT00338377 in the NCI website for specific exclusion criteria. Patients 18 years and older were enrolled in the clinical trial IRB-approved protocol 2009-0471 for the CXCR2 genetically modified TIL protocol. Refer to clinical trial NCI-2014-02655 and NCT01740557 in the NCI website for specific exclusion criteria. All patients have granted a written informed consent.

### Reagents

Human recombinant interleukin-2 (IL-2) (Proleukin™) was generously provided by Prometheus Therapeutics & Diagnostics. GMP-grade soluble anti-CD3 antibody (OKT3 clone) and Retronectin were obtained from Centocor Ortho Biotech and Takara Bio, respectively. The GMP-grade retroviral supernatant was produced by the Indiana University Vector Production Facility (IUVPF). Briefly a pMSGV1-CXCR2 retroviral construct was used to transfect PG13 cell line to produce viral supernatant. A high titer producing clone was isolated and used to generate a master cell bank (MCB). The MCB was extensively tested for sterility, presence of replication competent virus (RCR) and the stability of the insert. Upon passing all the testing, the MCB was used by IUVPF to produce one large lot of viral supernatant to suffice cell production for a clinical trial. The final GMP-grade viral supernatant is stored at −80°C.

### Isolation and Expansion of TIL from Metastatic Melanoma Tumors

Tumor-infiltrating lymphocytes were cultured from tumor fragments. Briefly, metastatic melanoma tumor samples were cut into 1–3 mm^2^ fragments and placed in complete TIL culture media (TIL-CM) with 6,000 IU/mL IL-2 in culture treated 24-well plates for a period of 3 to 5 weeks, as previously described (11).

### Generation of a cDNA Construct Expressing Human CXCR2

The retroviral vector pMSGV1, previously approved for clinical use, was used in this study (12, 13). The vector was generously provided by Dr. Steven A. Rosenberg. Human CXCR2 was amplified by PCR from human cDNA (purchased from ATCC) and cloned into the NcoI and EcoRI sites of the pMSGV1 vector. The CXCR2 construct was further confirmed by DNA sequencing. To detect CXCR2 protein expression, 293 cells were transfected with the DNA constructs and expression was confirmed by Flow Cytometry. The pMSGV1-CXCR2 construct was sent to IUVPF for GMP-grade retroviral supernatant production.

### Viral Transduction of PBMC and TIL

Frozen PMBC and TIL were thawed, and TIL were allowed to rest for a period of 1 to 2 days (day −5) at a concentration of 1.5 × 10^6^/mL in a tissue culture treated 24-well plate containing TIL-CM and 6,000 IU/mL of IL-2. Nunc 24-well plates were coated with anti-CD3 (OKT3) at up to seven different concentrations (0, 3, 10, 30, 100, 300, and 1,000 ng/mL). Sterile PBS (Dulbecco’s phosphate-buffered saline; Invitrogen) was used as a diluent for the OKT3 coating at 1 mL/well. An additional plate coated with 30 ng/mL anti-CD3 was prepared for the non-transduced (NT) control on day −1. The anti-CD3 coated plates were stored at 2–8°C until further use. Additionally, 24-well non-tissue culture treated plates were coated with Retronectin diluted at a concentration of 20 µg/mL using PBS (1 mL/well) and stored at 4°C until further use.

After the resting period (day −3), TIL were harvested, adjusted at a concentration 1.5 × 10^6^/mL (TIL-CM + 6,000 IU/mL IL-2) and 1 mL was distributed per well of OKT3-coated plates after washing the free antibody using PBS. For experiments using PBMC, the cells were activated using the same conditions except for the resting period which was omitted. Cells were activated for either 24 or 48 h under standard cell culturing conditions (37°C, 5% CO_2_) until the transduction step. On the day of the virus transduction (day −1), the Retronectin-coated plates were blocked with 2.5% human serum albumin in PBS for 30 min to an hour at room temperature followed by repeated washes in PBS. One milliliter of freshly thawed viral vector supernatant was added to each well of the Retronectin-coated plates. The plates were immediately centrifuged at 2,000 *g* for 2 h at a preheated temperature of 32°C. During the centrifugation of the virus, OKT3-activated TIL (for 24 or 48 h) were collected and adjusted to concentration ranging between 0.8 and 1.2 × 10^6^/mL. After removing the supernatant postcentrifugation, 1 mL of cell suspension was added to each well of the vector-coated plates. Finally, the plates were centrifuged at 1,000 *g* for 10 min and incubated at standard conditions (37°C, 5% CO_2_) until the Rapid Expansion Protocol (REP) initiation on the next day (day 0).

### REP Modified for the Expansion of Transduced TIL

Activated and gene-modified TILs were further expanded using a modified version of the REP (14, 15). The CXCR2-transduced TIL or the control OKT3-activated but non-transduced TIL were put in culture with pooled allogeneic irradiated PBMC feeder cells at a ratio of 1 TIL to 200 feeders and 6,000 IU/mL IL-2 on day 0 of the modified REP. The rest of the rapid expansion was carried out as previously described (2, 11).

### Flow Cytometry

PBMC or TIL were stained with fluorochrome-conjugated monoclonal antibodies (CD3, CD4, CD8, and CXCR2 from BD Bioscience) in FACS Wash Buffer (Dulbecco’s phosphate buffered saline 1× with 1% bovine serum albumin) for 30 min on ice for surface staining. Dead cells were excluded using AQUA live/dead dye from Invitrogen. For intracellular staining of active caspase-3, cells were fixed and permeabilized using Cytofix/Cytoperm (BD Bioscience) and stained with cleaved anti-caspase-3 (BD Bioscience) on ice as well. Acquisition of stained cells was done using BD FACSCanto™ II and analyzed using FlowJo software (Tree star).

### Tumor-Infiltrating Lymphocyte-Mediated Killing Using Autologous or HLA-A-Matched Tumor Lines

Killing of autologous or HLA-A-matched melanoma tumor cells (target cells) by gene-engineered TIL postexpansion was visualized by flow cytometry to measure the level of activated caspase-3 in target cells (16). To assess the class I dependency of killing, target cells were incubated for 3 h at 37°C with 80 µg/mL of either MHC class I blocking antibody W6/32 or the IgG2a-matching isotype control (eBioscience).

## Results and Discussion

### Assessment of Optimal Incubation Time and Plate-Bound Anti-CD3 Concentration for Efficient Transduction of CXCR2 and REP of TIL

**Figure 1:**
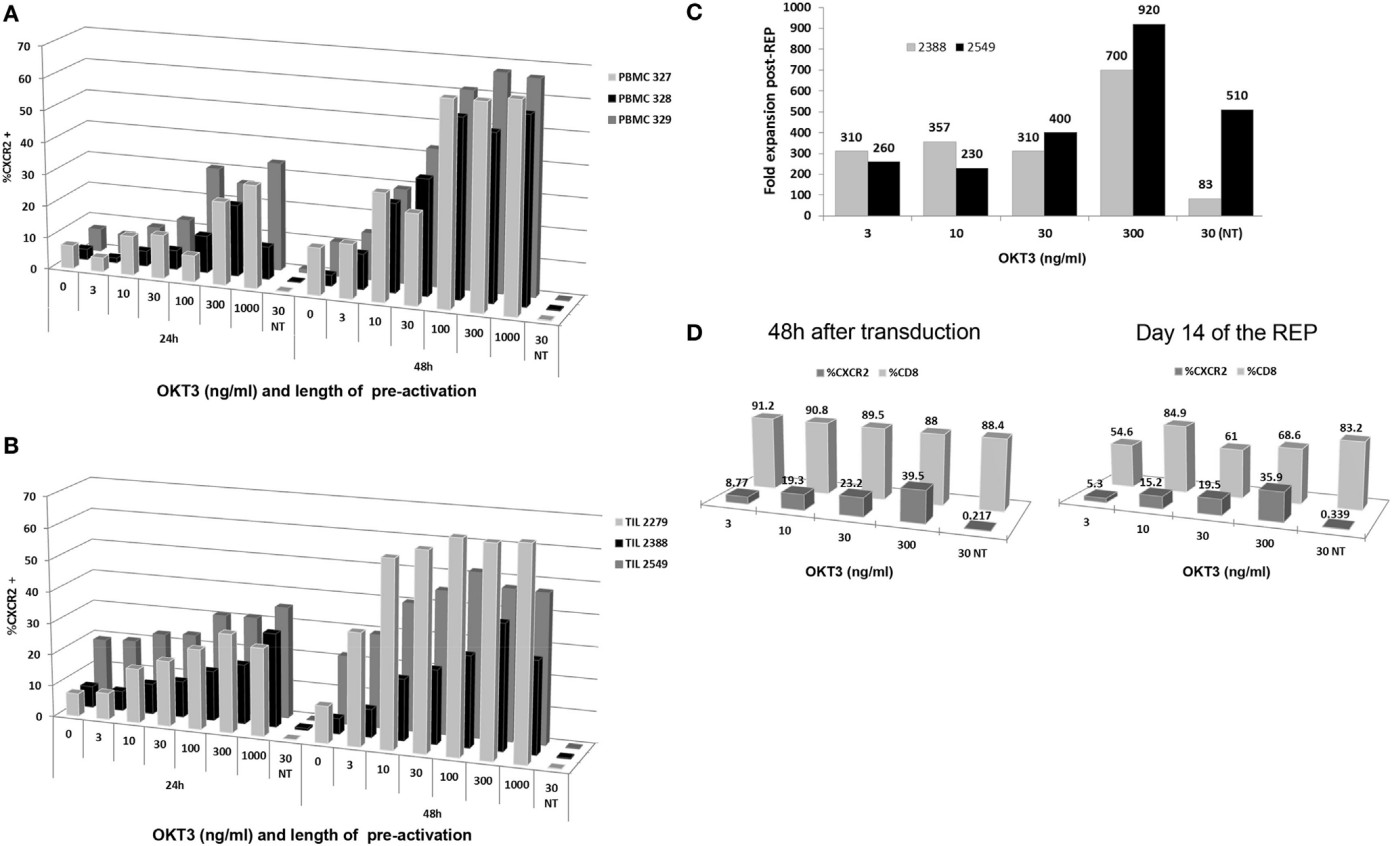
Optimization of time and antibody concentration for T cell activation prior to transduction. **(A)** Peripheral blood mononuclear cell (PBMC) transduction efficiency following 24 and 48 h plate-bound anti-CD3 (OKT3) activation at different concentrations of antibody. **(B)** Tumor infiltrating lymphocyte (TIL) transduction efficiency following 24 and 48 h plate-bound anti-CD3 activation at different concentrations of antibody. **(C)** Fold expansion post-Rapid Expansion Protocol (post-REP) based on different concentrations of anti-CD3 antibody used for plate-bound activation **(D)** Transduction efficiency and CD3^+^CD8^+^ T cell percentages obtained following plate-bound anti-CD3 activation at different concentration of antibody at 48 h after transduction and day 14 of the REP.

To develop a clinically relevant, GMP-friendly and efficient transduction method for TIL, we initially tested various concentrations of plate-bound anti-CD3 to activate cells as well as two different time points (24 and 48 h) of activation preceding the transduction step. The idea was to activate and transduce the cells prior to the REP to minimize the amount of plates used for transduction while still getting a large expansion of transduced cells at the end of the process. Initially, we tested the different conditions using normal donor PBMC for proof of principle and reproducibility. PBMC were activated and later transduced with the CXCR2 viral construct at 24 or 48 h postactivation. As shown in Figure 1A, the percentage of viable CXCR2^+^ lymphocytes at 48 h post-transduction increased accordingly with the increasing concentration of plate-bound anti-CD3 used for activation. Both time points showed this trend, but a higher percentage of CXCR2 transduction was observed after 48 h of OKT3 activation prior to transduction (*n* = 3). However, the percent transduction tended to reach a plateau at concentrations of anti-CD3 over 100 ng/mL for the 48 h time point.

To transpose this method to TIL, three lines were used to assess the transduction within the same concentration parameters and time points. As shown in Figure 1B, the efficiency of CXCR2 transduction increased with the concentration of anti-CD3 used for activation at both 24 and 48 h. However, as observed with the PBMC, a higher percentage of CXCR2 transduction was detected after 48 h of anti-CD3 activation, plateauing between 300 and 1,000 ng/mL of OKT3 concentration (Figure 1B). It is crucial to note that activation-induced cell death (AICD) does set a limiting target concentration for the use of plate-bound anti-CD3, especially with TIL since the majority are effector/effector memory cells whom have already been activated in the tumor or the surrounding microenvironment. Thus, the higher percentage of CXCR2 transduction at 48 h of activation and the plateauing tendency from 300 to 1,000 ng/mL, led us to choose the lowest optimal concentration of plate-bound anti-CD3 (300 ng/mL) for the activation of TIL pretransduction.

After transduction, two TIL lines were selected to further confirm a successful expansion of TIL using a modified REP approach. As described before, the REP usually includes an initial anti-CD3 stimulation, which occurs on day 0 with the addition of the irradiated feeder cells used as an expansion platform. Given the 48 h plate-bound anti-CD3 activation that TIL undergo prior to viral transduction, we eliminated the further use of anti-CD3 in the REP and replaced it by addition of 6,000 IU/mL of IL-2 on day 0 of the REP. This modification protects the TIL against AICD, which could be caused by repeated stimulations of the TCR, while preserving the beneficial growth stimulation provided by the feeder cells. The results at day 14 post-REP, shown in Figure 1C, demonstrate an upward trend in fold expansion from 3 to 300 ng/mL anti-CD3 activation pretransduction with a maximal fold expansion of 700 and 920 at the 300 ng/mL concentration (Figure 1C). Thus, even if lower anti-CD3 doses may deliver similar transduction efficiency, there is an important improvement in the yield of TIL obtained using 300 ng/mL anti-CD3 antibody, as opposed to lower doses. The non-transduced (NT) TIL control, stimulated with plate-bound anti-CD3 concentration of 30 ng/mL but omitting the transduction, proves that the viral transduction of the cells does not impair the TIL expansion since the expansion was comparable or improved in the transduced TIL as compared to the NT TIL for both TIL lines (Figure 1C).

Since our past experience with TIL ACT demonstrated the clinical relevance of a high percentage of CD8^+^ cells within the TIL infusion product, we assessed the percentage of CXCR2-transduced TIL in the CD8^+^ TIL population (2). As shown in Figure 1D, there was no significant changes in percentage of CD8^+^ TIL at 48 h post-transduction and 14-day post-REP. Thus, this demonstrates that the pre-activation of TIL at any of the tested concentrations (3 to 300 ng/mL) confers no disadvantage to the growth of CD8^+^ TIL. Nonetheless, a predictable change in percentage of CXCR2-transduced CD8^+^ TIL was observed in an upward trend with a maximal efficiency at 300 ng/mL (Figure 1D).

Altogether, it was concluded that activation of TIL with a concentration of 300 ng/mL plate bound anti-CD3 for a period of 48 h prior to transduction yielded the highest fold expansion and percentage of CXCR2 transduction in TIL while retaining a similar CD8^+^ percentage profile. These results established the conditions for activation and transduction of TIL prior to moving into the REP process.

### Modification in the REP Combined with Virus Transduction Does Not Affect the Proliferative Capacity of Genetically Modified TIL While Maintaining New Gene Expression

**Figure 2:**
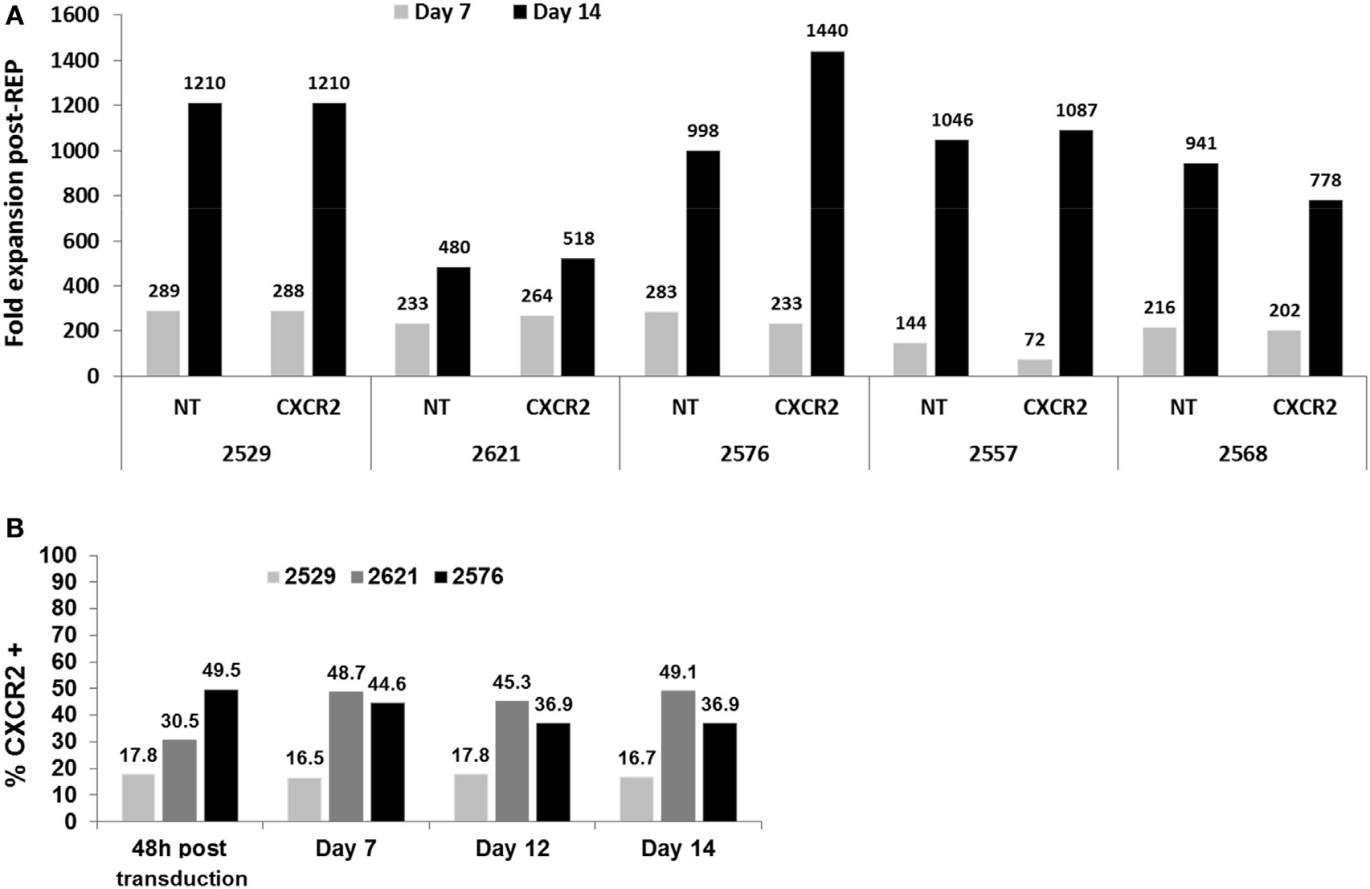
Fold expansion comparison of transduced tumor-infiltrating lymphocyte (TIL) post-Rapid Expansion Protocol (post-REP) and transduction preservation. **(A)** Fold expansion at days 7 and 14 of the REP for both CXCR2-transduced and non-transduced TIL lines. **(B)** Percentage of CXCR2 expression in CXCR2-transduced TIL at 48 h after transduction, days 7, 12, and 14 in REP.

As mentioned previously, to avoid over stimulation of TIL by activating them with OKT3 2 days prior to transduction and once again 1 day post-transduction (in the REP), we proceeded to modify the “traditional” REP, which we have been using in clinical TIL expansions. Traditionally, on day 0 of the REP, the TIL are added to an expansion platform constituted of the feeder cells loaded with anti-CD3. In our modified version, activated and transduced TIL are only added to the feeder cells in presence of high dose of IL-2, without further TCR stimulation (no anti-CD3). To investigate any potential changes this modification may have on the final TIL product, as well as any potential effect of the transduction on the REP process, we first looked at fold expansion between transduced and NT TIL with five lines at days 7 and 14 of the REP (Figure 2A). In general, a similar fold expansion was observed in both transduced and NT TIL lines on day 7 and between days 7 and 14. From our extensive experience with TIL therapy, our expectation/criteria for a successful REP is to obtain 100-fold expansion at day 7 followed by a further 10-fold increase over the second week for a total of 1,000-fold (acceptable range 500–3,000). All the expansions conducted with transduced or NT TIL met those criteria (Figure 2A). Thus, we conclude that neither the modification in the REP nor the transduction itself will abrogate the infusion of a respectable number of genetically modified TIL.

Furthermore, to assess any changes in transduction percentage during the REP, three TIL lines were selected to study the expression of CXCR2 by flow cytometry throughout the rapid expansion process. Four time points post-transduction (48 h, days 7, 12, and 14) were selected. As demonstrated in Figure 2B, none of the expanded TIL lines showed any noteworthy loss of CXCR2 expression in percentage at any point. Therefore, the pre-activation and transduction of TIL prior to the REP with our optimized concentration of plate bound anti-CD3 showed no disadvantageous change in fold expansion and a stable expression of CXCR2 throughout the REP. In a clinical TIL production setting, one could think of sampling cells on day 12 of the REP and use this sampling to establish the percentage of transduction prior to a fresh infusion on day 14 of the REP, avoiding the need for same day flow cytometry assessment to establish transduction efficiency on the day of infusion prior to releasing the cells for infusion.

### Genetically Modified TIL Retain Their Cytotoxic Capacity

**Figure 3:**
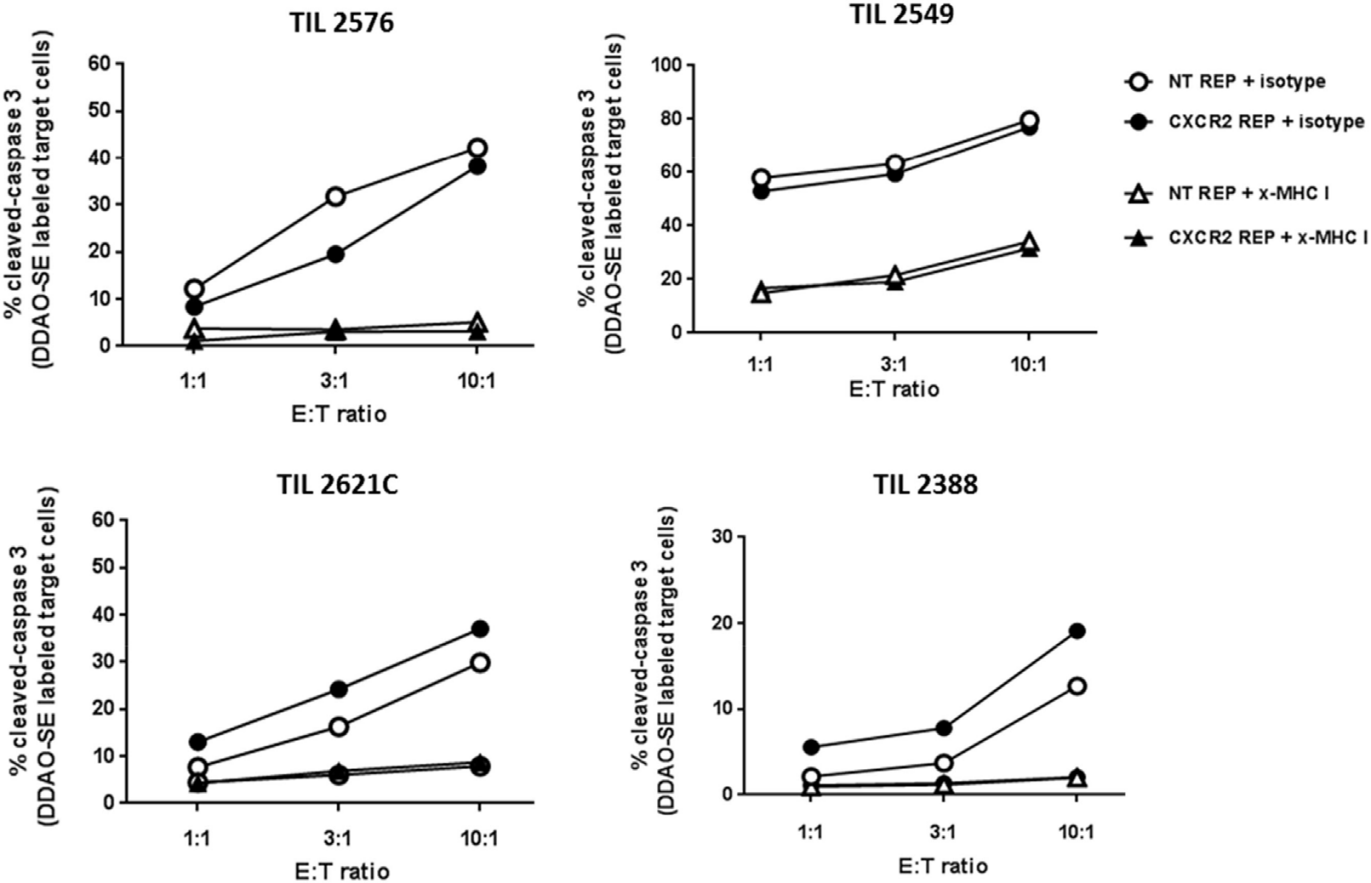
Retained cytotoxic capacity of virally transduced tumor-infiltrating lymphocyte (TIL). Percentage of cleaved caspase-3 in target tumor cells (autologous or HLA-A matched) after coculturing at three different effector to target tumor cell ratios for CXCR2-transduced TIL (black circles) and non-transduced TIL (white circles). MHC class I blockage was used as control (x-MHC I) and shown in triangles in the legend.

A crucial factor to demonstrate in our method of viral transduction of TIL and propagation is the preservation of effector traits such as their lytic capability. Transduced and NT TIL were rapidly expanded and their cytotoxic capacity was assessed by measurement of active cleaved caspase-3 in tumor target cells by flow cytometry. Accordingly, tumor targets (autologous or HLA-A-matched tumor cell lines) were labeled with DDAO-SE dye and cocultured at three different ratios of effector:target cell (1:1, 3:1, and 10:1). As shown in Figure 3, increased percentage of active caspase-3 in tumor targets was observed at higher E:T ratios. There was no noteworthy difference in tumor target killing between CXCR2-transduced and NT TIL. Furthermore, the blocking of MHC-I on tumor targets impaired the killing ability of both CXCR2-transduced and NT TIL, supporting a lytic capability depending directly on epitope recognition following presentation by an MHC class I molecules. Hence, this demonstrated that there is no significant change in cytotoxic capacity of TIL post-REP following viral transduction.

Altogether, our method preserves expansion potential, CD8^+^ T-cell frequency and lytic capabilities of TIL, leading us to the presumption that the TIL product will be suitable for infusion, presenting the enviable qualities associated with the success of TIL ACT. The last step was to show that this technique can reproducibly yield high numbers of gene-modified clinical-grade TIL products for patients’ treatment.

### Clinical Generation and Expansion of CXCR2-Transduced TIL

**Figure 4:**
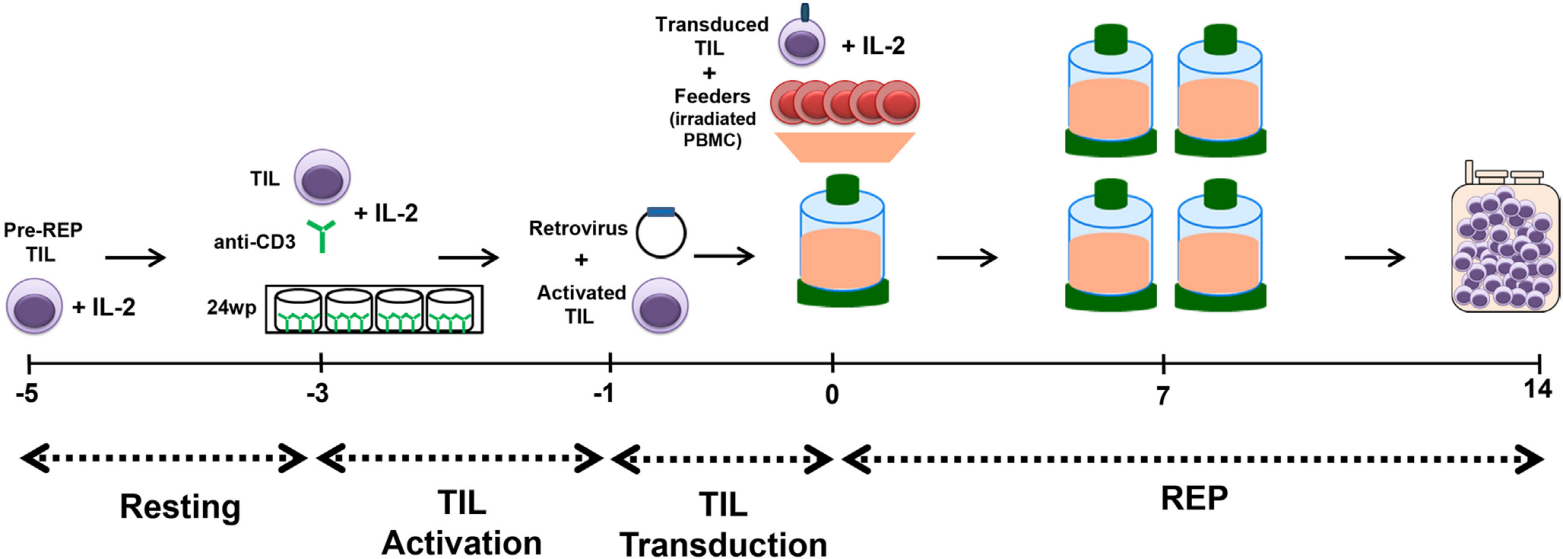
Schematic of Rapid Expansion Protocol (REP) with transduction of tumor-infiltrating lymphocyte (TIL). TIL are thawed on day −5 and allowed to rest for 2 days. The resting period is followed by activation of the TIL with plate-bound anti-CD3 on day −3 and transduction is performed on day −1. Finally, the REP process is followed from days 0 to 14 with the exception that we omit the initial anti-CD3 stimulation on day 0. Genetically modified TIL are harvested and infused fresh on day 14 of the REP.

This optimized method for TIL genetic modification was elaborated and is now used for the manufacturing of TIL for two protocols at MDACC. This process is performed in a large-scale, GMP-compliant setting. The schematic presented in Figure 4 illustrates the established workflow for the production of genetically modified TIL: TIL are thawed on day −5 and allowed to rest for 2 days, which is followed by activation with plate-bound anti-CD3 on day −3 and transduction is performed on day −1. Finally, the REP process is followed from days 0 to 14 with the exception that we omit the initial anti-CD3 stimulation on day 0.

**Figure 5:**
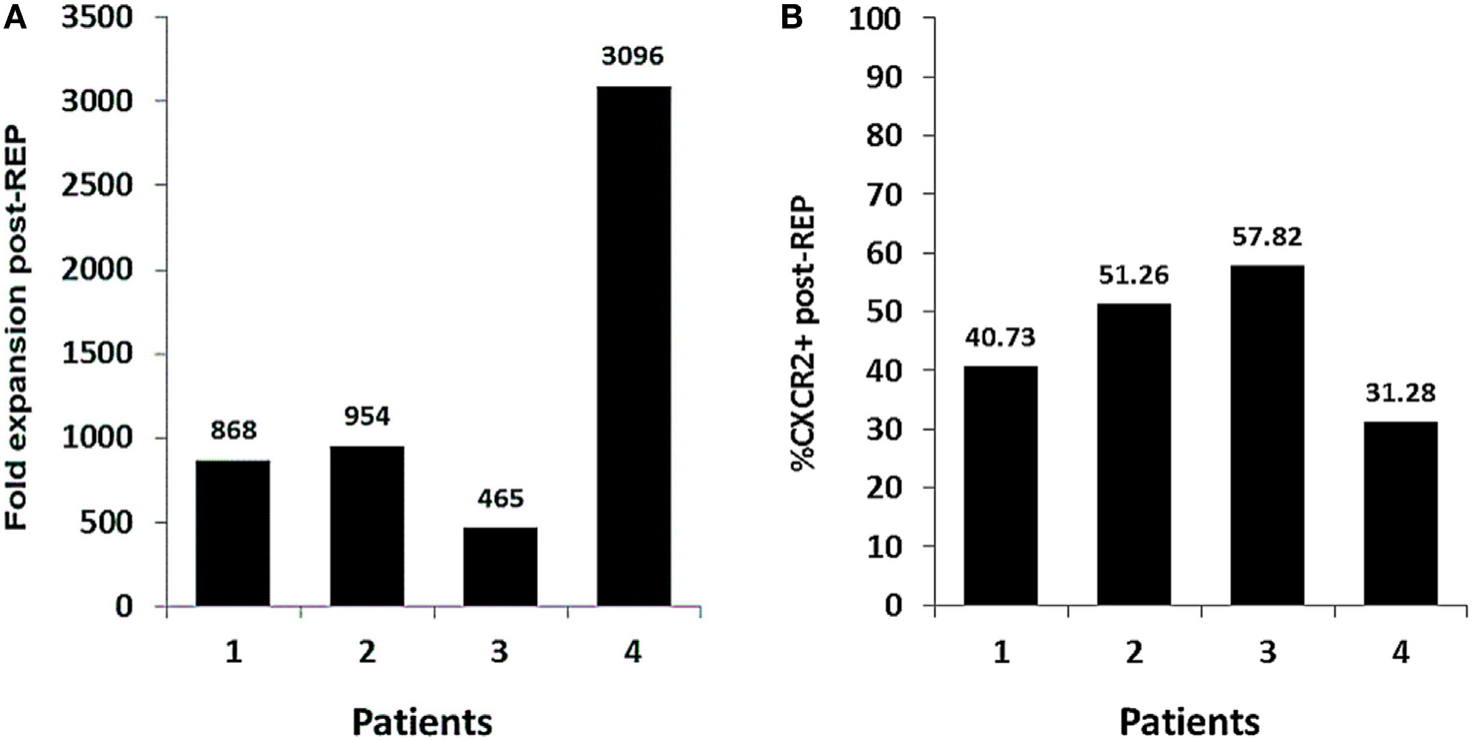
Fold expansion and transduction efficiency of Good Manufacturing Practices (GMP)-grade, genetically modified tumor infiltrating lymphocyte (TIL) in the context of clinical production. **(A)** Fold expansion CXCR2-transduced TIL lines at day 14 of the clinical Rapid Expansion Protocol (REP) (*n* = 4). **(B)** Percentage of CXCR2 expression in CXCR2-transduced TIL at day 14 of the clinical REP (*n* = 4).

The optimization of our method has proven effective for the expansion of genetically modified TIL. Herein, we present four TIL lines that were successfully transduced to express CXCR2 and expanded for the treatment of patients with metastatic melanoma. Even though patient to patient variability in the REP fold expansion was observed, all TIL lines were successfully expanded and infused (range from 465- to 3,096-fold expansion) (Figure 5A). The transduction levels of CXCR2 at infusion in all of the lines were ranging from 31.28 to 57.82% of expression (Figure 5B). Overall, the number of CXCR2-transduced TIL infused to each patient ranged from 0.8 to 19.3 billion cells. However, the trial design involves the transfer of an equal number of TIL transduced with a control gene (truncated version of NGFR); therefore, patients were infused with a total number of virally transduced TIL ranging between 1.6 and 38.6 billion cells. In addition, for each patient, a smaller parallel TIL expansion with non-genetically modified TIL was setup to maximize the number of cells infused given a maximal infusion cap of 150 billion TIL. Since this trial includes the infusion of three different TIL products (CXCR2-transduced TIL, NGFR-transduced TIL, and NT TIL), patients were infused with a range between 7 and 143.8 billion TIL. This range is comparable to what we previously published in our first generation of TIL ACT (2).

A fifth patient was enrolled in this trial but could not be infused due to the lack of TIL growth observed on day 7 of the REP. The starting TIL product on day −5 of the transduction contained high levels of CD3^+^CD8^−^CD4^−^ TIL (37.5%) and did not expand. To our knowledge, this “double negative” population could be considered a regulatory population that may interfere with the proliferative capacity of TIL in the REP (17). This population is rarely observed in our pre-REP TIL cultures but should be avoided with this technique. We would also speculate that higher levels of NK cells in the pre-REP product should also be accounted for. NK cells will not be activated on day −3 of the procedure, as they are not responsive to an anti-CD3 stimulation, and could create the illusion of a suboptimal TIL expansion.

Our novel method to generate and expand clinical-grade, genetically modified TIL permits us to infuse higher numbers of transduced cells than previously described in other studies where the transduction is carried within the REP process (9, 10). Although the transduction after 4 days in the REP yields high transduction efficiency, the presence of a 200:1 ratio of irradiated feeders in the culture coupled with a higher number of T cells to be plated at low density for efficient contact with the virus during transduction presents unique challenges. Attempts to transduce the TIL prior to the REP had not been adopted in part because restimulating TIL with anti-CD3 a few days after an initial stimulation prior to transduction may trigger AICD and lead to poor expansion. We have devised a process in which TIL can be efficiently genetically modified and expanded to clinically relevant numbers. We handle a maximum of two to four plates on transduction day which yields up to several billions of gene-modified TIL for infusion. Thus, this method could be easily incorporated into similar protocols to produce GMP-grade genetically modified TIL in environments where space is restricted. It is our hope that these “functionally enhanced” TIL will convey lasting tumor control benefits to the patients. The streamlining of the genetic modification of TIL will pave the way for new trials and will lead to significant improvement in outcome to TIL therapy.

## Ethics Statement

This study was carried out with the recommendations of the University of Texas MD Anderson Cancer Center Institutional review board with written informed consent from all subjects in accordance with the Declaration of Helsinki. The protocol was approved by the University of Texas MD Anderson Cancer Center Institutional review board.

## Author Contributions

MF was involved in conception and design of the study and methodology, experiments and acquisition of data, clinical production, as well as manuscript writing and review. RT participated in experiments and acquisition of data, clinical production, and manuscript writing and review. CH participated in experiments and acquisition of data as well as writing and review. RR, SM, MZ, SW, CT, and WP were involved experiments and data acquisition. OF was involved in clinical production and data acquisition. AG, ST, EF, and AW were involved in clinical production of TIL. RA was involved in patient care. PH was involved with conception and design of the study as well as patient care. CB participated in conception and design of the study and methodology, experiments and acquisition of data, and manuscript writing and review. All authors have read and approved the final manuscript.

## Conflict of Interest Statement

The authors declare that the research was conducted in the absence of any commercial or financial relationships that could be construed as a potential conflict of interest.

## Abbreviations

TIL, tumor-infiltrating lymphocytes; ACT, adoptive cell therapy; GMP, Good Manufacturing Practice; PBMCs, peripheral blood mononuclear cells.
